# Structure Characterization and Impact Effect of Al-Cu Graded Materials Prepared by Tape Casting

**DOI:** 10.3390/ma15144834

**Published:** 2022-07-11

**Authors:** Jianian Hu, Ye Tan, Xuemei Li, Youlin Zhu, Guoqiang Luo, Jian Zhang, Ruizhi Zhang, Yi Sun, Qiang Shen, Lianmeng Zhang

**Affiliations:** 1 State Key Laboratory of Precision Blasting, Jianghan University, Wuhan 430100, China; hujianian1@outlook.com; 2 Chaozhou Branch of Chemistry and Chemical Engineering Guangdong Laboratory, Chaozhou 521000, China; lmzhang@whut.edu.cn; 3 National Key Laboratory for Shock Wave and Detonation Physics, Institute of Fluid Physics, China Academy of Engineering Physics, Mianyang 621900, China; lixuem@caep.cn (X.L.); zhangrz1991@gmail.com (R.Z.); 4 State Key Laboratory of Advanced Technology for Materials Synthesis and Processing, Wuhan University of Technology, Wuhan 430070, China; youlinzhu51@gmail.com (Y.Z.); zhangjian178@whut.edu.cn (J.Z.); sunyiwhut@163.com (Y.S.); sqqf@263.net (Q.S.)

**Keywords:** Al-Cu composite, graded materials, interface reflection principle, acoustic impedance, gas gun experiment

## Abstract

With the need of developing new materials, exploring new phenomenon, and discovering new mechanisms under extreme conditions, the response of materials to high-pressure compression attract more attention. However, the high-pressure state deviating from the Hugoniot line is difficult to realize by conventional experiments. Gas gun launching graded materials could reach the state. In our work, the corresponding Al-Cu composites and graded materials are prepared by tape casting and hot-pressing sintering. The microstructure and the acoustic impedance of the corresponding Al-Cu composites are analyzed to explain the impact behavior of Al-Cu graded materials. Computed tomographic testing and three-dimension surface profilometry machine results demonstrated well-graded structure and parallelism of the graded material. Al-Cu GMs with good parallelism are used to impact the Al-LiF target at 2.3 km/s using a two-stage light-gas gun, with an initial shock impact of 20.6 GPa and ramping until 27.2 GPa, deviating from the Hugoniot line.

## 1. Introduction

The research of shock wave physics has developed rapidly, and various high-pressure loading methods have been used to load materials such as explosives, metals, and organics to study their equations of state under shock loading conditions [1,2,3,4,5]. Nowadays, high-pressure loading and in situ transient techniques are used to develop new materials, explore new phenomena, and discover new mechanisms under extreme conditions [6,7]. The results of these studies are amazing under Hugoniot state or hydrostatic pressure, such as carbon-based hydride material room-temperature (288 K) superconductivity [8], methane gas decomposing into metal hydrogen and diamond [9], and phase transformations of plutonium nuclear material [10]. However, these new discoveries correspond to the state equation of material in the shock state (Hugoniot line is a line connecting the stress-specific volume states of materials under different shock states) or quasi-static state, which is the state of incomplete stress, specific volume and specific internal energy [11,12,13,14].

To obtain a more complete state equation of the material, it is very important to use the high-pressure loading method to obtain the state of the material deviating from the Hugoniot line [15,16,17,18]. Nevertheless, the phenomena deviating from the Hugoniot line has obtained little attention. On the one hand, the test stress-strain curve is close to the ideal elastoplastic time test, which usually has an approximation shock compression. On the other hand, many researchers believe that the strain rate effect of (metal) materials is only sensitive to the logarithm of variability, so a certain amount of strain rate change in the experiment will not have a significant impact on the test results [19]. However, in recent years, as stated in the MaRie project [20], one of the key contributions of high-pressure science is through the creation of extreme conditions of pressure and temperature, which has led to new discoveries. In the pressure-temperature phase diagrams, it is possible to explore new insights that cannot be achieved by traditional methods based on various physical processes, such as the melting line of metallic materials. The material dynamics strategic plan published by the Sandia National Laboratories laboratory in 2017 [21,22] also mentioned that the shock-quasi-isentropic loading platform is about to be deployed to detect phase boundaries to reach pressure, temperature, and strain rate regions that are difficult to reach with existing platforms, to correct the large deviations of existing material models, and to determine the time-dependent material response characteristics in strength and phase transitions (solid-solid, solid-liquid, liquid-solid) during dynamic compression.

Quasi-isentropic loading experiments are confirmed to achieve a state deviating from the Hugoniot line [23,24]. However, it is very difficult to realize the shock-quasi-isentropic loading process, and the related loading process is extremely difficult to achieve by Z machine and laser impact. At present, W-Cu graded materials (GMs) have been prepared for quasi-isentropic loading at the high-voltage side, and Mg-Cu graded materials are used for the low-voltage side [25,26,27]. However, Mg is the hcp phase, it is prone to phase transition under high pressure, and it is difficult to realize the shock-quasi-isentropic process in the low pressure range. The Fcc Al structure is more stable than the hcp Mg structure under high-pressure loading [28], therefore, in this paper, Al-Cu graded materials were made to realize phase transition under relative low pressure range.

There are many investigations on the preparation methods of graded composite materials, including gradient powder metallurgy [29], diffusion welding [30], explosive welding [31], laser additive manufacturing [32], and tape-cast powder metallurgy [33]. Tape-cast powder metallurgy is a promising method for preparing GMs in the high-pressure experiment, owing to the accurate controlling scale, density and thickness of the GMs, which are essential parameters in the quasi-isentropic loading experiments to explore the phase boundary [33]. By adding organic additives to the mixed powder in different proportions, cast plain sheets with a uniform thickness of 50–100 μm are obtained by casting. Then, the GMs are densified and sintered. By controlling the microstructure and mechanical behavior of the graded material in the dynamic experiment, a corresponding quasi-isentropic curve can be obtained as a ramp loading with an initial shock loading. 

In this paper, Al-Cu graded materials were prepared and verified by theoretical calculations and experiments systematically. Al-Cu composites and corresponding Al-Cu GMs are prepared by tape casting and hot pressing sintering. The physical properties of the graded structures are predicted from the monolithic properties, such as density and acoustic impedance. Computed tomographic testing and three-dimension surface profilometry machine results demonstrated good graded structure and parallelism of the graded material. The GMs are used to impact Al-LiF target at 2.3 km/s using a two-stage light-gas gun, and the experimental pressure curve is measured by fiber-optic displacement interferometer (DISAR). The pressure is analyzed based on the interface reflection principle, with an initial shock impact to 20.6 GPa, ramping until 27.2 GPa, which deviates from the Hugoniot line.

## 2. Experiment Procedure

**Figure 1:**
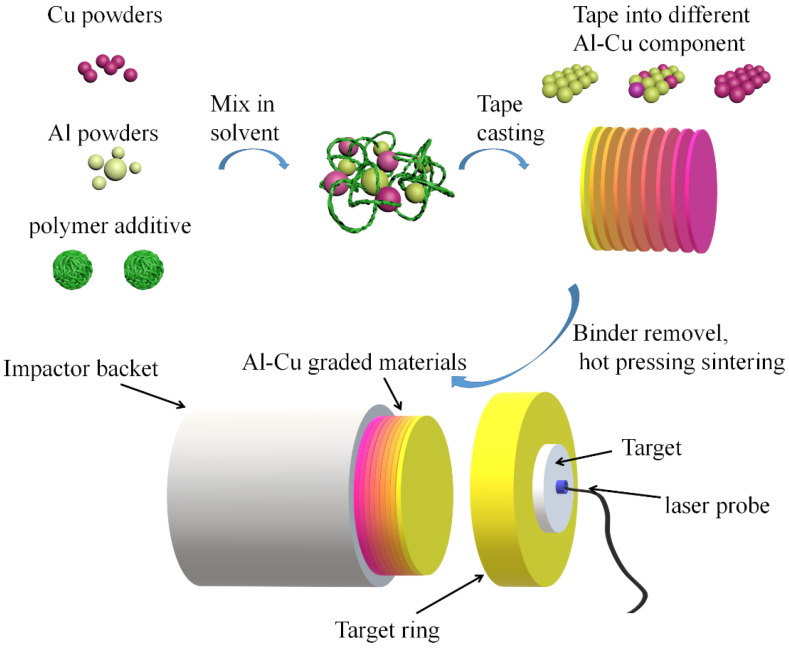
Schematic diagram of the experimental process.

Figure 1 shows the schematic diagram of the experimental process. First, Al powders with a diameter of 5 μm and Cu powders with a diameter of 5 μm were mixed with 3% polyvinyl butyral (PVB) and alcohol in a zirconia ball mill at a speed of 240 rmp for 12 h to obtain Al-Cu casting slurries with different components. By controlling the blade height of the casting machine, the thickness of the cast film after drying can be controlled. The dried Al-Cu cast film is cut into preset diameters mold, and the cut Al-Cu casts are stacked in sequence according to the designed graded structure [34]. Then, the whole mold and Al-Cu casts inside are heated under 450 °C to remove the PVB in a vacuum atmosphere. After PVB removal, the mold and Al-Cu green body inside is heated to a sintering temperature of 500 °C. 

The acoustic impedance and thickness distribution of the graded materials was essential for the impacting experiment, however, the acoustic impedance and thickness was determined by the phase composition, density and graded structure, so in our study, the carbon component of the Al-Cu samples were determined by the elemental analyzer (Vario EL cube, Elementar, Germany). X-ray photoelectron spectroscopy (VG Multilab 2000, Bruck, Germany) was used to characterize the component of the surface of Al-Cu samples. Field-emission scanning electron microscopy (Quanta-250, FEI, America), energy-dispersive spectrometry (EDS), and high-resolution transmission electron microscopy (JEM-2100F, JEOL, Japan) were used to evaluate the microstructure and component of the Al-Cu composite. The X-ray diffraction (Utima II, Rigaku, Japan) patterns and microstructure of different Al-Cu samples are characterized to analyze the phase distribution, then the density (*ρ*) is obtained by the Archimedes drainage method, and the acoustic velocity (*v*) can be characterized by the reflected wave test method. The acoustic impedance (*Z*) can be obtained by *Z* = *ρ × v*.

Then, the Al-Cu GMs are prepared based on the equation *Z*_(*x*)_ = *Z*_0_ + A(*x*/d)^2^, where *Z*_0_ is the initial acoustic impedance of the first layer in the graded materials, *Z*_(*x*)_ and *x* represents the acoustic impedance and the thickness of nth layer in the graded materials, and d is the whole thickness of the graded materials. By this design criterion, quasi-isentropic loading effect is better. The flatness of Al-Cu graded material is characterized by the three-dimensional optical profiler (ST400). The three-dimensional X-ray reconstruction of CT is used for the Al-Cu GMs non-destructive testing, including parallelism measurement and internal defect detection.

After that, the Al-Cu GMs with a diameter of 23 mm is used as impactors to be launched at high velocity for a dynamic experiment on a two-stage gas gun [28]. The graded materials were launched by two-stage gas gun with 11 MPa N_2_ in the launch tube, and 0.15 MPa N_2_ in the pump tube. The high-pressure gas in launch tube activates impactors to compress the gas in pump tube until membrane rupture pressure is 15 MPa, then the compressed gas in pump tube activates the graded materials to impact the target. Sample data from a target consisting of a 2.0 mm thick Al baseplate tamped with a LiF window is obtained. The particle velocity history is measured at the sample window Al-LiF interface using a Displacement Interferometer (DISAR), which was based on the optical Doppler effect, all single-mode fibers are used to transmit optical signals, and optical interference mixing technology is used to obtain the continuous velocity information of the object. The LiF window is a transparent material used to convert the particle velocity signal of the target into an optical signal for optical fiber detection.

## 3. Result and Discussion

**Figure 2:**
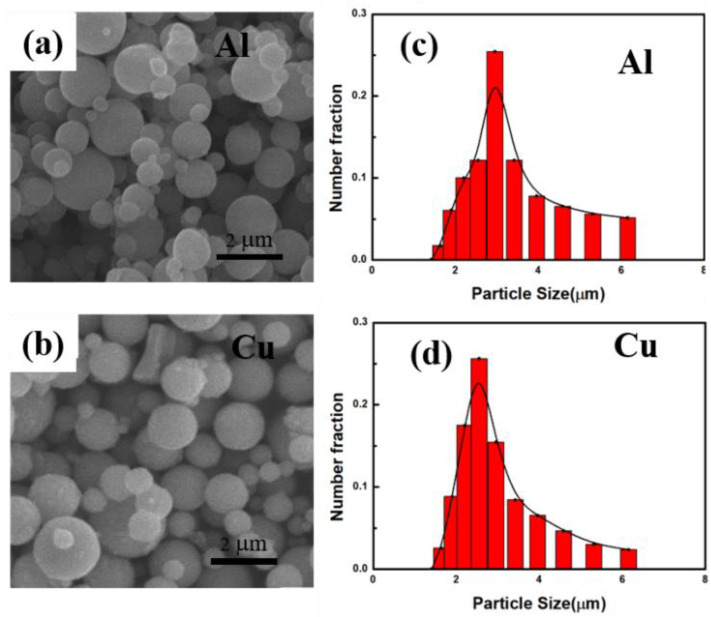
(**a**,**c**) SEM image and particle size distribution of the original Al powder; (**b**,**d**) SEM image and particle size distribution of the original Cu powder.

Figure 2 shows the SEM image and particle size distribution of the raw material Al powder and Cu powder. It can be seen that the raw Al powders and Cu powders are spherical. Besides, the particle size distribution of Al and Cu both range from 2 to 3 μm. The particle size distribution promotes uniform phase distribution in the preparation of gradient materials.

**Figure 3:**
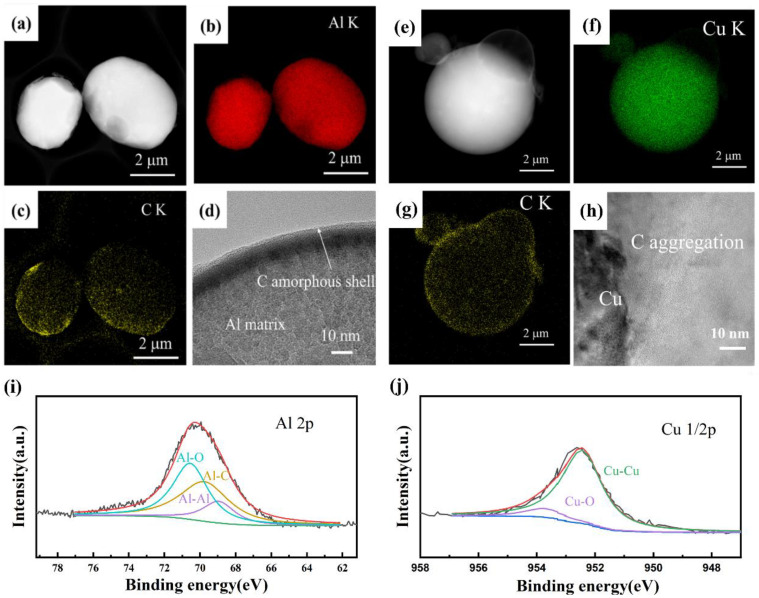
(**a**) TEM image of Al powders after polymers removal, (**b**,**c**) Al and C element distribution of Al powders after polymers removal, respectively. (**d**) HRTEM image of C distribution on Al particle surface, (**e**) TEM image of Cu powders after polymers removal, (**f**,**g**) Cu and C element distribution of Al powders after polymers removal, respectively, (**h**) HRTEM image of C distribution on Cu particle surface, (**i**,**j**) XPS results of Al powders and Cu powders after polymers removal, respectively.

Figure 3 shows the TEM images and EDS results of the Al and Cu powders after PVB removal. From Figure 3a, the results suggested that spherical Al powder of the initial raw material becomes ellipsoid, due to the ball milling. The EDS results of Al and C element in Figure 3b,c indicate that there is a layer of C on the surface of the powder. From the HRTEM results in Figure 3d, it can be seen that the C layer is uniformly coated on the surface of the Al powder. Figure 3e shows the TEM images of the Cu powder after polymers removal. The results suggested that the spherical Cu powder of the initial raw material is not deformed after ball milling. The EDS results shown in Figure 3g show that the C layer on the surface of the powder has agglomerated, indicating that the adsorption capacity of amorphous pyrolytic C on Cu is poor. The HRTEM results in Figure 3h indicate that the amorphous pyrolytic C is distributed around the Cu powder particles. Besides, XPS results of Al powders and Cu powders after polymers removal shown in Figure 3i,j indicate good bonding of Al-C and poor bonding of Cu-C, respectively. The XPS results are analyzed based on the work function shifting method [35,36], the work function (WF) of the samples is measured as 3.85 eV, and all other referring spectra are shifted, accordingly.

**Figure 4:**
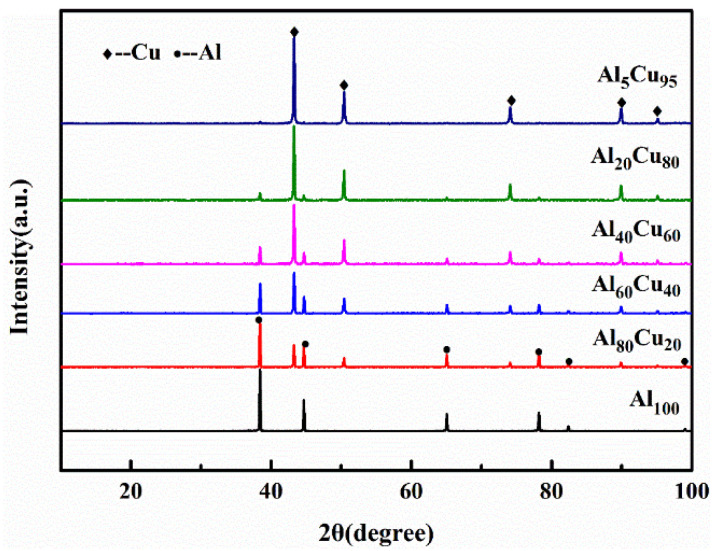
The XRD patterns of Al-Cu powders with different compositions after polymers removal.

Figure 4 shows the XRD results of the Al and Cu powders with different components after polymers removal. Only Al and Cu diffraction peaks are clearly observed in all samples, indicating that no reaction is activated during the polymers removal process between Al and Cu powders. The XRD results of initial Al-Cu powder can be provided to analyze whether intermetallics will generate during the sintering process.

**Figure 5:**
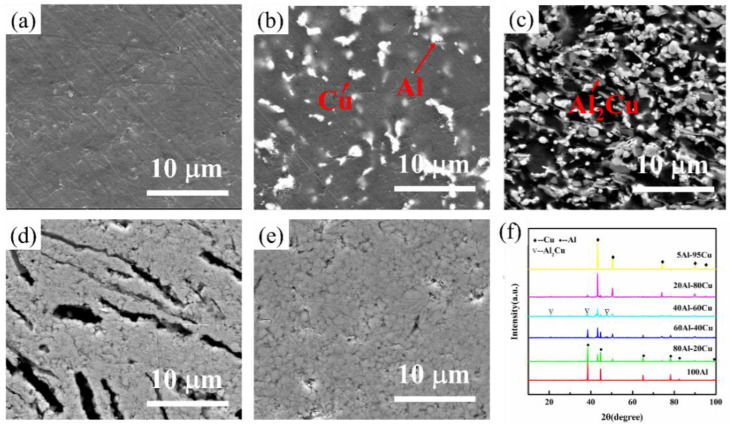
The microstructures of Al-Cu samples with different Al content. (**a**) 100 wt.% Al; (**b**) 80 wt.% Al; (**c**) 60 wt.% Al; (**d**) 5 wt.% Al; (**e**) 0 wt.% Al, (**f**) the XRD pattern of individual Al-Cu composites with different compositions.

The microstructures of Al-Cu samples sintered under 500 °C with different components are shown from Figure 5a–e. From the results, the distribution of Al and Cu particles can be observed in all figures without obvious pores, suggesting high-quality densification and good dispersion of Al and Cu particles. In addition, some grey Al_2_Cu intermetallic compounds can be observed on the interface of Al and Cu particles, corresponding to the XRD results shown in Figure 5f. From the results, Al and Cu diffraction peaks can be clearly observed in all samples, however, Al_2_Cu intermetallic compound peaks can be observed in Al/Cu samples. The XRD peaks suggest that the main phases in samples are Al and Cu phases, besides, Al_2_Cu intermetallic compound is generated. 

**Figure 6:**
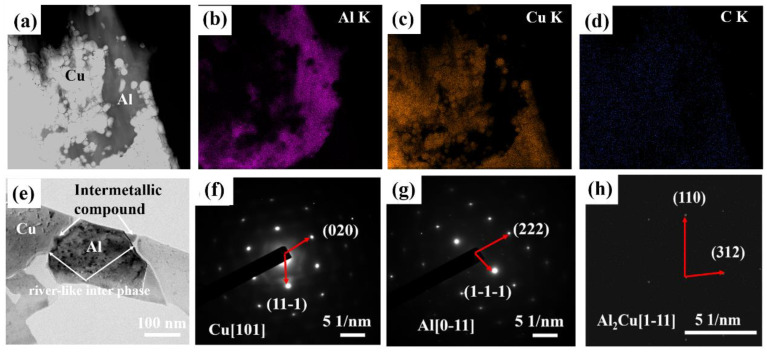
(**a**) TEM image of the Al-Cu composite under sintering temperature of 500 °C, (**b**–**d**) EDS results of Al, Cu, C elements, respectively, (**e**) higher magnification TEM image of the Al-Cu composite, and (**f**–**h**) are the selected area electron diffraction patterns of the corresponding area in (**a**), respectively.

In order to prove the phase composition of the intermetallic compound, we carried out transmission characterization of the Al-Cu composite. The TEM image of the Al-Cu composite and EDS results of Al, Cu, and C elements are shown from Figure 6a to Figure 6d, respectively. Figure 6e shows the higher magnification TEM images of the Al-Cu composite, and the results suggest that there are mainly four phases in the Al-Cu composite material. In order to determine the phases of the three phases, we carried out a selected area electron diffraction (SAED) analysis on the different phases. From the results of the selected area electron diffraction, we can see that the white phase is Cu. The black phase is the Al phase, the gray phase between Al and Cu is the Al_2_Cu phase, and the amorphous carbon still exists on the grain boundary of Al-Cu interface. However, the adsorption properties of carbon on the surface Al and Cu lead to the fluidity of carbon under the sintering temperature 500 °C, according to the literature [36]. Voids will appear between grain boundaries between Al and Cu, thus not hindering the reaction between Al and Cu.

To determine the C elements and Al_2_Cu intermetallic compound, the C content in Al-Cu compound is determined by the CHONS analyzer, which is shown in a list, and the Al_2_Cu content is determined by the SEM images. The phase distribution of the Al-Cu composite with different components is shown in Table 1, and the physical properties of different phases in Al-Cu composite is shown in Table 2.

**Figure 7:**
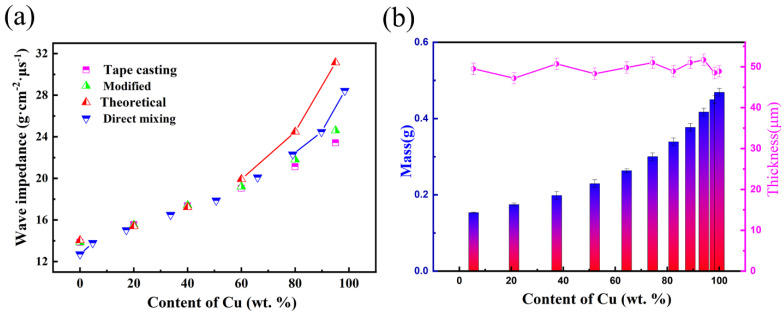
(**a**) Comparison of experimental wave impendence and designed value; (**b**) layer thickness of the tape for GMs by mass measurement.

To evaluate the effect of the microstructure of Al-Cu composites on acoustic impedance properties, the comparison of experimental acoustic impedance and designed value of tape casting—hot pressing consolidated composites and directly mixing sintering compacted composites—are shown in Figure 7a. The impedance experimental value is accurate to ±4% for the designed value of Al-Cu composites. The results suggest that the generation of the Al_2_Cu intermetallic compound did not affect the experiment value, owing to the wave impendence of Al_2_Cu being the same as the value of Al and Cu mixture in the ratio of generating the Al_2_Cu. The layer thickness of the tape for GMs is shown in Figure 7b. The thickness of the tape is controlled by the mass of that, based on the density obtained from the individual monolayer materials and the constant mass reduction of removing the polymer additive. The average thickness of the tape is 50 ± 2.5 μm when controlling the error below 5%. The consolidated layer thickness of the GMs can be modified based on the accurate measurement of mass before removal of the PVB.

**Figure 8:**
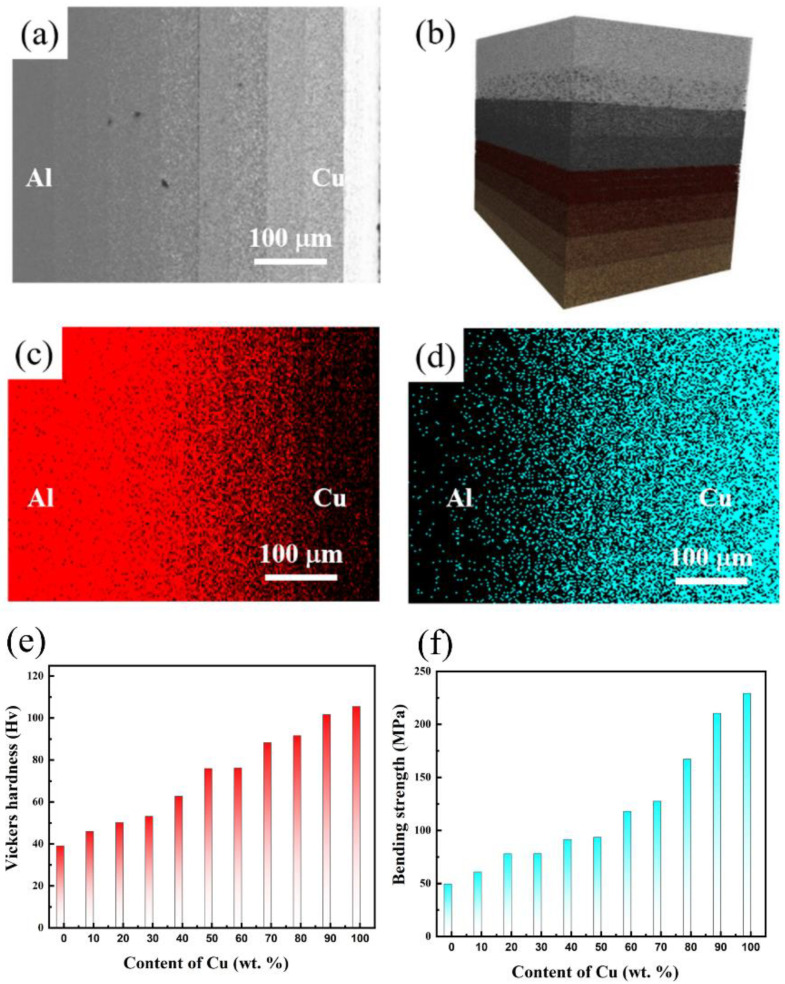
Microstructures of Al-Cu GMs (**a**) 2D view; and (**b**) 3D view. The element distribution in EDS results in (**c**) Al; (**d**) Cu and (**e**,**f**) Vickers hardness and bending strength of Al-Cu composite with different components, respectively.

The 2D and 3D microstructures of Al-Cu GMs are shown in Figure 8a,b, respectively. The results suggest the graded structure and distribution of Al and Cu particles without obvious pores in the Al-Cu GMs, suggesting high-quality densification and good dispersion of Al and Cu particles. The EDS results of Al and Cu distribution are shown in Figure 8c,d, respectively, suggesting a typically graded microstructure of Al and Cu distribution. The gradual component increment results in a variety of Vickers hardness and bending strength of Al-Cu composite with different components, which are shown in Figure 8e,f, respectively.

**Figure 9:**
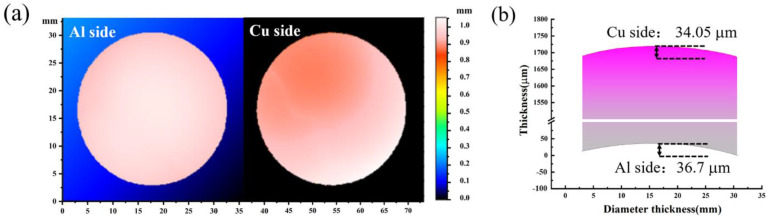
(**a**) Three-dimensional profiler and flatness of Al-Cu GMs; (**b**) two-dimensional view of the GMs.

The designed curve is chosen and processed into Al-Cu GMs. The surface parallelism of Al-Cu GMs characterized by a three-dimensional profiler is shown in Figure 9. The large difference in the flatness of the GMs indicates the internal large thermal expansion coefficient difference and internal stress. From the two-dimensional height difference map of the Al surface and Cu surface in Figure 9a, it can be seen that the different colors in the figure represent different height differences. In the two-dimensional plan view, we can see that the Al surface is low in the middle and high around it, which is concave. The two-dimensional height difference diagram of the Cu surface shows that the Cu surface is convex. The flatness analyzed from Figure 9a is shown in Figure 9b. The results suggest the flatness of the Al surface is 36.7 μm, while the flatness of Cu surface is 34.05 μm. Good surface parallelism ensures the accuracy of the data in the gas gun experiment.

**Figure 10:**
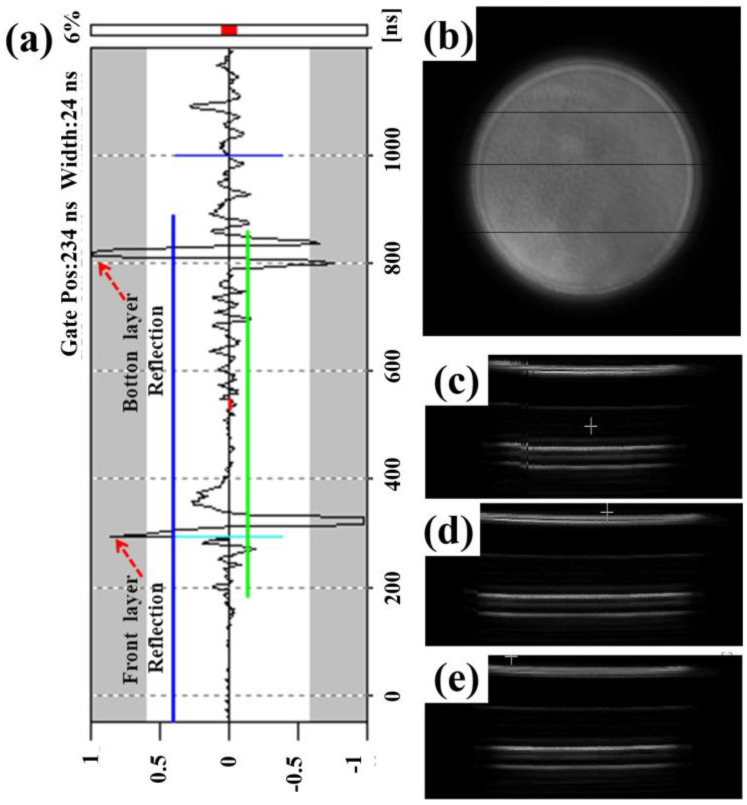
(**a**) Ultrasonic nondestructive reflection wave signal of the GMs; (**b**) internal flat structure of the GMs; (**c**–**e**) internal graded structure of the GMs.

To have a better internal observation of the Al-Cu GMs microstructure prepared by tape casting, ultrasonic nondestructive testing is used to characterize the Al-Cu GMs in Figure 10a. The graded structure is observed by modifying the ultrasonic reflection wave signal. Figure 10b presents an internal flat view of the GMs, suggesting high density of the GMs without pores. To observe a detailed graded structure in the internal of the GMs, three cross-section structures are shown in Figure 10c–e, and the parallel layers suggest that the GMs exhibit a multi-layer structure with good parallelism between the Al-Cu layers. 

**Figure 11:**
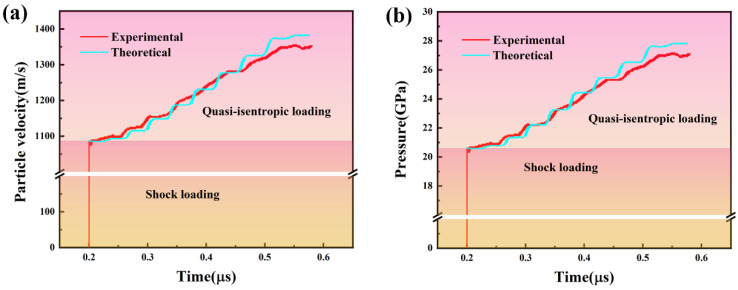
(**a**) Particle velocity results of the GMs impactor experiment; (**b**) pressure results of the GMs impactor experiment.

Figure 11a shows the particle velocity history of the Al-Cu GMs launched to impact a 2 mm Al target with a LiF window. The particle velocity in the Al target can be transferred from the initial data characterized by the laser probe. The particle velocity results suggest a typical quasi-isentropic loading features, ramping from 1087 m/s to 1360 m/s. So, the pressure was analyzed by wave system in the process of GMs flyer shooting and is shown in Figure 11b. Initially, the particle velocity curve shows an initial shock impact within 0.1 ns, and the platform of the pressure of 20.6 GPa corresponds to the structure of the pure Al layer in the GMs. A smooth wave platform is ramping up from 20.6 GPa to 27.2 GPa until the final platform, which is related to every layer of the GMs until the Cu layer. The pressure result obtained from the gas gun experiment has a good fitness with the initial designed pressure-time curve. Uncertainty between the designed and experimental pressure-time data corresponds to the designed and experimental impedance-thickness data, which have a relative low value below 4%, duo to the impedance change by the sintering interfacial diffusion in the Al-Cu GMs. 

**Figure 12:**
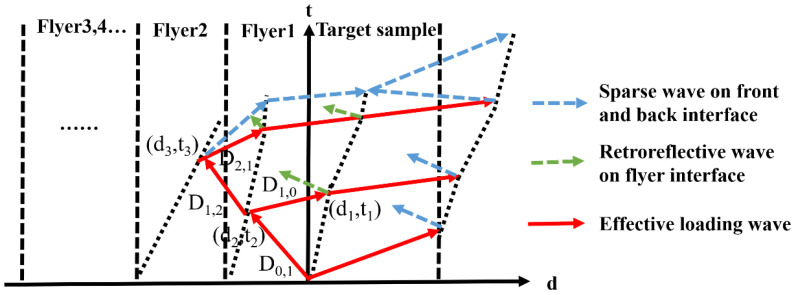
Schematic diagram of the graded material waves propagation system.

The analysis of the Al-Cu GMs impact experiment is based on the wave system in the process of GMs flyer shooting, which is shown in Figure 12 [37]. The solid line is the shock wave propagation path, the dotted line is the interface before the collision, the line is the interface after the collision, and the dot-dash line is the propagation path of the sparse wave. The free surface particle velocity analysis method has been discussed and compared with experimental data, proving its reliability. 

Supposed the GMs travel under-speed w, and after GMs collide with the stationary target, the shock waves will propagate in the GMs. From the shock wave relationship, the direct equation can be described as follows:

(1a) $$D_{0,1}=C_{0,0}+\lambda_{0,0}u_{0,1},$$

(1b) $$P_{0,1}=\rho_{0,0}D_{0,1}u_{0,1},$$

(1c) $$-D_{1,1}-w=-C_{1,0}+\lambda_{1,0}\left(u_{1,1}-w\right),$$

(1d) $$P_{1,1}=\rho_{1,0}\left(-D_{1,1}-w\right)\left(u_{1,1}-w\right),$$

where *D*_m,n_ is the speed of the shock waves (m indicates the number of layers of shock wave propagation, and the target is 0 layers; *n* indicates how many times the layer has undergone compression). Based on the boundary conditions, *P*_1,1_ = *P*_0,1_, *u*_1,1_ = *u*_0,1_, the particle velocity *u* and pressure *P* inside the flyer 1 and target can be solved, then Z = *P*/*u* can be solved. When the shock wave *D*_0,1_ is transmitted to the interface between the flyer 1 and the flyer 2, since the impact impedance of the flyer 1 is smaller than that of the flyer 2, then flyer 2 transmits a shock wave D_2,1_ and reflects a shock wave *D*_1,2_ into flyer 1, then reloads flyer 1. The relationship is listed as follows:

(2a) $$D_{1,2}-u_{1,1}=C_{1,1}+\lambda_{1,1}\left(u_{1,2}-u_{1,1}\right),$$

(2b) $$P_{1,2}-P_{1,1}=\rho_{1,1}\left(D_{1,2}-u_{1,1}\right)\left(u_{1,2}-u_{1,1}\right).$$

According to the continuous conditions of the interface, the state of the flyer 1 and the flyer 2 after being impacted can be obtained. When the shock wave *D*_1,2_ continues to propagate to the interface of the flyer 1 and the sample, the reflection and transmission of the wave will also occur. The transmitted shock wave will load the sample again. If there is a flyer with a higher impact impedance behind the flyer 2, *D*_2,1_ will continue to reflect a shock wave after passing to the rear surface of the flyer 2, and this shock wave will pass through the flyer 2 and the flyer 1 to the sample, thus creating third shock loading in the target. By analogy, when there are *n* layers of flyer, the sample will be compressed *n* times.

Assuming that the thickness of the sample is *d*_0_, the thickness of the flyer 1 is *d*_1_, and the thickness of the flyer 2 is *d*_2_, from the shock wave relationship, the equations are directly written as follows:

(3a) $$d_{0}=t_{1}D_{1,1},$$

(3b) $$d_{1}=t_{2}\left(w+D_{1,1}\right),$$

(3c) $$x_{2}=-D_{1,1}t_{2},$$

(3d) $$t_{3}=\left(D_{1,2}t_{2}-x_{2}\right)/\left(D_{1,2}-u_{1,1}\right),$$

(3e) $$x_{3}=u_{1,1}t_{3}.$$

The thickness of the flyer 1 can be solved based on the above equations, by analogy, when there are *n* layers of the flyer, the thickness of the flyer *n* can be solved. The theory has been validated against LLNL data [24], and the data fit between theory and experiment is very good, so related theoretical calculation methods are also used in this study. This experimental technique and theory can be used to explore the phase transition properties of Bi near the impact melting line in the range 20–27 GPa [38].

## 4. Conclusions

In this paper, a novel gradient material for quasi-isentropic loading is prepared, characterized, and evaluated.

The designed Al-Cu composites and GMs are prepared by tape casting. Both Al and Cu powders contain C layer after heat treatment, besides, the Al_2_Cu intermetallic compound is observed from the microstructure. However, the acoustic impedance and thickness of the Al-Cu sample are controlled with an error lower than 5%. The Al-Cu GMs are processed with a low flatness of 34.05 μm on the Cu side and 36.7 μm on the Al side. The typical internal obvious graded structure was detected by ultrasonic NDT and 3D CT.

The Al-Cu graded materials were designed by an acoustic impedance quadratic curve, and the theoretical pressure curve was calculated based on reflection principle. Then, the Al-Cu graded materials were launched on a two stage gas-gun to impact an Al-LiF window. The pressure curve obtained from the impact experiment is a smooth wave platform ramping up from 20.6 GPa to 27.2 GPa until the final platform, fitting the designed curve well with an error below 4%, deviating from the Hugoniot line.

## Figures and Tables

**Table 1:** The phase distribution of the Al-Cu composite with different components.

Cu (wt.%)	0	10	20	30	40	50	60	70	80	90	100
C	0.08	0.43	0.46	0.48	0.29	0.53	0.79	0.61	0.73	0.84	0.73
Al_2_Cu	0.00	2.30	4.81	7.60	11.62	16.67	10.50	7.33	5.32	2.65	0.00
Al	0.00	8.71	17.30	25.73	33.58	40.69	53.83	65.59	76.5	87.8	99.2
Cu	99.92	88.56	77.43	66.18	54.51	42.11	34.88	26.46	17.4	8.70	0.00

**Table 2:** The physical properties of different phases in the Al-Cu composite.

Phase	Density (g cm^−3^)	Acoustic Speed (km/s)	Acoustic Impedance (g cm^−2^ μs^−1^)
Carbon	2.203	4.45	9.80
Al	2.712	5.33	14.45
Cu	8.924	3.91	34.89
Al_2_Cu	4.065	4.95	20.12

## Data Availability

The data that support the findings of this study are available from the corresponding author upon reasonable request.
